# Treatment of Chills and Fever

**Published:** 1875-10

**Authors:** D. P. Duncan

**Affiliations:** Waynesboro, Georgia


### TREATMENT OF CHILLS AND FEVER.

By D. P. DUNCAN, M.D., Waynesboro, Ga.

It has been my intention for several months to give to the profession my treatment for chills and fever, which is the result of four years' experience in a county where the physician has a glorious field for experimenting in that disease. Two cases which I have recently relieved are conclusive evidence to my mind that we need go no further to find a specific for this terrible malady.

Facts are what we should strive for in physic; not theories, which are only fit for the individual more in search for repute than the promotion of science. And facts are what the practitioner of medicine wants; and if capable, as he should be, to perform the duties of his sacred calling, he can easily adapt the treatment to the peculiar constitutional differences and temperaments of his patients. He has not the time nor the inclination to read a dozen pages for a fact; and feeling the truth of my assertion, I shall give you in as few words as possible my treatment. Every morning before breakfast I direct the patient to take a pill containing 2 grs. of quinine, 2 grs. Vallet's mass, and 1-60 of a gr. of strychnia. In addition I give three times a week a pill composed of—

| | |
|---|---|
| ℞.—Podophylln, | gr. $\frac{1}{4}$. |
| Comp. Ext. Colocynth, | gr. j. |
| Tr. Ext. Aloes, | gr. j. |
| Ipecac, | gr. $\frac{1}{2}$. |
| Ext. Hyoscyamus, | gr. ij. |

At the same time keeping the region of both liver and spleen tender by the use of croton liniment; and if the spleen be considerably enlarged, as is generally the case in chills of long standing, I use bromide potassium in from 10 to 20 gr. doses upon retiring to bed. The above followed for 30 days will invariably put an end to this most troublesome and insidious of all affections, from the fact that we are completely unnerved without being able to appreciate it, and rendered more susceptible in this weakened condition to the ravages of disease, which the system in perfect working order would be successfully able to combat.

I use the first and second combinations as a prophylactic in the malarial region, and have yet to see the case where it has not been effective, if followed according to directions.

---

"Augustus, dear," said she, tenderly pushing him from her as the moonlight flooded the bay-window where they were standing, "I think you had better try some other hair-dye; your mustache tastes like turpentine."

